# Home Energy-Efficiency Retrofits

**DOI:** 10.1289/ehp.1103621

**Published:** 2011-07-01

**Authors:** Brent Stephens, Ellison M. Carter, Elliott T. Gall, C. Matt Earnest, Elizabeth A. Walsh, Diana E. Hun, Mark C. Jackson

**Affiliations:** National Science Foundation Integrative Graduate Education and Research Traineeship (IGERT) Program in Indoor Environmental Science and Engineering, The University of Texas at Austin, Austin, Texas, E-mail: stephens.brent@mail.utexas.edu; Oak Ridge National Laboratory, Oak Ridge, Tennessee; Lennox International Inc., Carollton, Texas

In the February 2011 issue of *EHP*, Manuel (2011) took an important look at some potential adverse health implications of home energy retrofits. Here, we further discuss the complexity of possible indoor environmental concerns and encourage incorporation of comprehensive homeowner education campaigns in weatherization programs.

The reduction of air infiltration by air sealing is a common energy retrofit measure (McCold et al. 2008). Several field studies of weatherized homes have reported average reductions in air leakage of 13–40% (Berry and Brown 1994; Judkoff et al. 1988), although the impact of weatherization on actual air exchange rates and indoor pollutant concentrations is poorly understood. Moreover, studies have seldom evaluated the effects of weatherization on low-income groups or vulnerable populations (e.g., asthmatic or elderly), although occupants in low-income residences are at higher risk for many indoor environmental hazards (Evans and Kantrowitz 2002), and some population subgroups may also be disproportionately affected by indoor air pollution (Hun et al. 2009).

Although some research exists on the impact of weatherization on indoor concentrations of combustion products, radon, and moisture, other indoor pollutants deserve attention. For example, Logue et al. (2011) identified nine priority indoor air pollutant hazards in U.S. residences, which, among others, have been associated with a wide range of both chronic and acute health effects (e.g., benzene, 1,4-dichlorobenzene, formaldehyde, naphthalene, particulate matter < 2.5 µm in aerodynamic diameter). Moreover, reducing air exchange rates in residences will likely increase indoor concentrations of reactive pollutants and the probability of chemical reactions occurring between them indoors (Weschler and Shields 2000), generating by-products associated with respiratory symptoms and asthma, such as low-molecular-weight aldehydes, dicarbonyls, and secondary organic aerosols (Jarvis et al. 2005). On the other hand, reductions in air infiltration should decrease penetration of outdoor pollutants, which is of particular importance in traditionally leakier low-income households (Chan et al. 2005) in neighborhoods with high outdoor air pollution. Thus, we urge the environmental health community to investigate the net effects of weatherization on a wide variety of indoor and outdoor pollutants and health outcomes.

Implementation of home energy retrofits also creates an opportunity to incorporate innovative, engaging homeowner education strategies to reduce both energy consumption and indoor environmental risks. Occupant behavior has a major influence on both energy consumption (Allcott and Mullainathan 2010) and indoor exposures to pollutants (Meng et al. 2005). Furthermore, many indoor air quality risks can be mitigated with relatively simple home behavior practices, such as using exhaust fans, avoiding toxic cleaning chemicals, and using appropriate air cleaners (Brugge et al. 2003). However, we have learned from research on household energy consumption that educational materials alone usually fail to alter behaviors (Charles 2009). Greater energy savings from home retrofits could be achieved by complementing homeowner education campaigns with regular feedback on energy use and economically motivational programs (Peschiera et al. 2010). Additionally, home walkthroughs with trained building specialists can identify energy-inefficient behaviors and appliances in conjunction with potential indoor environmental hazards. These and other behavior-change strategies to promote green and healthy housing should be made available to weatherization programs across the country, and their effectiveness should be assessed. Because home weatherization is currently a priority of the federal government, this is a crucial time to address these fundamental research questions and implement the findings nationwide.
